# Genetic Characterization, Antibiotic Resistance, and Virulence Genes Profiling of *Bacillus cereus* Strains from Various Foods in Japan

**DOI:** 10.3390/antibiotics13080774

**Published:** 2024-08-16

**Authors:** Marwa Nabil Sayed Abdelaziz, Mahmoud Gamaleldin Zayda, Aye Thida Maung, Mohamed El-Telbany, Tahir Noor Mohammadi, Su Zar Chi Lwin, Khin Zar Linn, Chen Wang, Lu Yuan, Yoshimitsu Masuda, Ken-ichi Honjoh, Takahisa Miyamoto

**Affiliations:** 1 Department of Bioscience and Biotechnology, Graduate School of Bioresource and Bioenvironmental Sciences, Kyushu University, 744 Motooka, Nishi-ku, Fukuoka 819-0395, Japan; marwanabil@agr.kyushu-u.ac.jp (M.N.S.A.); ayethidamaung@agr.kyushu-u.ac.jp (A.T.M.); mohamedeltelbany@agr.kyushu-u.ac.jp (M.E.-T.); suzarchilwin@agr.kyushu-u.ac.jp (S.Z.C.L.); khin.zar.309@s.kyushu-u.ac.jp (K.Z.L.); wangchen@agr.kyushu-u.ac.jp (C.W.); yuanlu930423@yahoo.co.jp (L.Y.); y.masuda@agr.kyushu-u.ac.jp (Y.M.); honjoh@agr.kyushu-u.ac.jp (K.-i.H.); 2 Department of Food Hygiene, Animal Health Research Institute (AHRI), Agriculture Research Center (ARC), Giza 12618, Egypt; 3 Department of Food Hygiene, Faculty of Veterinary Medicine, University of Sadat City, Sadat City 32897, Egypt; mahmoud.gamal@vet.usc.edu.eg; 4 Biology Department, San Diego State University, San Diego, CA 92182, USA; tnoormohammadi@sdsu.edu

**Keywords:** *Bacillus cereus sensu stricto*, multi-locus sequence typing analysis, multidrug resistance, β-lactam antibiotics, MALDI-TOF MS

## Abstract

*Bacillus cereus sensu stricto* is a foodborne pathogen that causes food poisoning. Their spore and biofilm-forming abilities persist in various environments and foods. This study investigated the prevalence, virulence, antibiotic resistance, and genetic diversity of *B. cereus s. s.* strains isolated from various food samples. Of 179 samples, 22.34% were positive for *B. cereus s. s.*, with significantly high detection rates in milk products and raw chicken meat. Forty strains were isolated from positive samples. Matrix-assisted laser desorption ionization/time of flight mass spectrometry analysis revealed nine distinct clusters and multi-locus sequence typing revealed 34 sequence types including 23 novel sequences, demonstrating high genetic diversity among the isolates. PCR analysis revealed that all the strains contained at least one toxin gene, but none contained the *cytK* gene. Antibiotic resistance tests revealed that all isolates were classified as multidrug-resistant, with high resistance levels, particularly to β-lactam antibiotics and vancomycin, but were susceptible to gentamicin. All isolates showed variations in biofilm formation. This study highlights the significant public health risk due to *B. cereus s. s.* and underscores the need for stringent monitoring and control measures in food production to manage antimicrobial resistance and ensure food safety.

## 1. Introduction

Many foodborne pathogens that pose substantial health threats are present in food processing plants. Among these pathogens, *Bacillus cereus sensu stricto* is widely distributed in soil, water, plants, animals, and humans, and easily enters the food processing chain. This poses a high risk of contaminating raw and processed foods, including cereals, vegetables, milk products, and meat. Consequently, *B. cereus s. s.* is a major food-related pathogenic bacterium [1,2].

*B. cereus s. s.* is a gram-positive, rod-shaped, aerobic or facultatively anaerobic, motile, spore-forming microorganism that causes foodborne illnesses in humans. This bacterium contributes to the production of two distinct types of toxins responsible for diarrhea and emetic syndromes [3]. The diarrheal type is associated with one or more enterotoxins produced in the small intestine: hemolysin BL (HBL), consisting of three genes of *hblA*, *hblC,* and *hblD*; nonhemolytic enterotoxin (NHE), encoded by *nheA*, *nheB*, and *nheC*; cytotoxin K (*CytK*); enterotoxin T *(bceT*); and enterotoxin FM (*EntFM*). The emetic type of food poisoning is induced by a heat-stable toxin produced in food called cereulide, which is encoded by the *ces* gene [4]. *B. cereus s. s.* is also associated with serious infections, such as pneumonia, bacteremia, endophthalmitis, osteomyelitis, and endocarditis [5,6]. Emetic syndrome is usually linked to the consumption of rice and pasta, whereas diarrhea is mainly transmitted through vegetables, dairy products, and meat [7]. Rice is a staple food worldwide, including in Japan, and has been linked to numerous *B. cereus*-related food poisoning incidents. *B. cereus* is often underreported in official epidemiological data due to surveillance systems varying between countries, leading to inconsistent reporting and outbreaks that may not be definitively linked to *B. cereus* because symptoms resembling those of *S. aureus* and *C. perfringens* make identification difficult. Additionally, many people do not ask for medical help due to mild symptoms, and contaminated food samples are often unavailable for testing. This complicates estimating the global impact and comparing data between countries [8]. Notable outbreaks occurred during a birthday party in Italy [9] and in a Belgian kindergarten [10], and 117 outbreaks occurred in the USA from 1998 to 2008 [11]. In Europe, two cases of foodborne illnesses with vomiting, likely caused by dairy products contaminated with *B. cereus* were reported in England in 1975 and Denmark in 1981. Large-scale outbreaks associated with dairy products occurred twice in the European Union in 2013 and in the United States between 1998 and 2008. In Japan, a significant foodborne illness outbreak occurred in 1990, in which 291 patients fell ill after consuming inadequate ultra-high-temperature sterilized (UHT) milk, where 277 experienced vomiting and 208 (71.5%) developed symptoms within six hours of consumption [12,13,14]. Cases of food poisoning due to *B. cereus*-contaminated processed rice-producing emetic toxins have also been reported [15], along with diarrheal food poisoning cases [7]. Additionally, *B. cereus* bacteremia and meningitis outbreaks have been linked to contaminated hospital linen [16,17] and medical supplies, such as catheters, causing bloodstream infections in children in Japan [18]. *B. cereus s. s.* has been associated with many outbreaks in Europe, Japan, and North America, ranging from 1% to 22% [19]. Moreover, *B. cereus s. s.* is ranked the second and third most dangerous foodborne pathogen in France and China, respectively [20,21]. According to the following facts, because raw milk is highly contaminated with *B. cereus*, there is concern that deaths of neonates due to *B. cereus* infection from milk-borne illnesses, such as infant formula or baby food, may occur. Infant infection with *B. cereus* has increased sharply recently, as reported by [22]. Consequently, ref. [23] recommended that the levels of this pathogen and its spores in dried milk infant formula should be minimized to less than 100 CFU/g [24]. *B. cereus* invasive infections in neonatal ICUs, potentially linked to the ingestion of contaminated milk, have been frequently suspected and investigated. Two studies that documented severe intestinal infections in preterm neonates associated with *B. cereus* suggested a possible role of contaminated, pooled breast milk, though this was not confirmed through strain typing [25,26]. Another study reported nine cases of *B. cereus* bacteremia in five neonatal resuscitation units (NRUs) within the APHP between August and December 2016 [27]. Recently, a study by [28] documented the first case of *B. cereus* bacteremia in a preterm infant, definitively linked to the consumption of contaminated breast milk through pulsed-field gel electrophoresis [29]. Fatal poisoning outbreaks are usually caused by the cytotoxic gene which has hemolysis and necrosis activities in cells [30].

Recently, more psychrotolerant *B. cereus s. s.* strains have been identified that can grow at temperatures as low as 7 °C, in addition to the optimal range of 25–40 °C. This is a major issue in minimally processed chilled foods. Mild processing preserves the organoleptic properties of food but allows the survival of spores, which can germinate and grow at refrigeration temperatures, producing toxins. The emergence of these psychrotolerant *B. cereus s. s.* strains is concerning, as they have adapted to thrive under low-temperature conditions [31].

Globally, antimicrobial resistance is increasing due to excessive or improper drug use. Treatment of foodborne illnesses, including those caused by *B. cereus s. s.*, typically involves antimicrobial treatment. However, the emergence of antibiotic-resistant *B. cereus s. s.* strains can lead to treatment failure. Therefore, monitoring the antibiotic resistance profile of *B. cereus s. s.* is crucial for understanding resistance patterns and developing effective treatment strategies [32].

Multi-locus sequence typing (MLST) is an unambiguous procedure for characterizing bacterial isolates using the sequences of seven housekeeping genes [33] in *B. cereus s. s.* MLST is widely used because of its accurate identification and ability to create comparative databases. This method is a valuable epidemiological tool for tracking the distribution and genetic relatedness of *B. cereus s. s.* strains [34]. 

To the best of our knowledge, no study has assessed the overall risk of *B. cereus s. s.* in Fukuoka, Japan. Therefore, this study aimed to investigate the prevalence of *B. cereus s. s.* in various foods, evaluate its antibiotic resistance profile, and identify virulence genes and the genetic polymorphisms in these isolates. In order to monitor alterations in the *B. cereus s. s.* antibiotic spectrum, results were compared with those of *B. cereus s. s.* strains isolated from food and food manufacturing facilities in Japan 20 years ago. 

## 2. Results

### 2.1. Prevalence of B. cereus s. s. among the Samples Tested

The findings indicated that 40 of the 179 samples tested were positive for *B. cereus s. s.* Specifically, 18 were from milk and its products, 9 were from raw chicken meat, 3 were from tofu, 6 were from cooked rice, and 4 were from soil and bedding. Only samples identified as containing presumptive *B. cereus* belonging to *Bacillus cereus sensu lato* (*n* = 89) on XBC agar were further streaked onto the *Bacillus cereus* rapid agar (BACARA) plate. Confirmatory tests were performed on positive BACARA isolates (*n* = 50). Positive BACARA isolates (*n* = 47) were presumptively identified as *B. cereus s. s.* and subjected to further confirmation of the presence of the *gyrB* gene by using polymerase chain reaction (PCR). The overall isolation rate from all samples was 22.34%, which represented 40 strains (Table 1).

### 2.2. Identification and Classification of B. cereus s. s. Isolates Using Matrix-Assisted Laser Desorption Ionization/Time of Flight Mass Spectrometry (MALDI-TOF MS)

**Figure 1:**
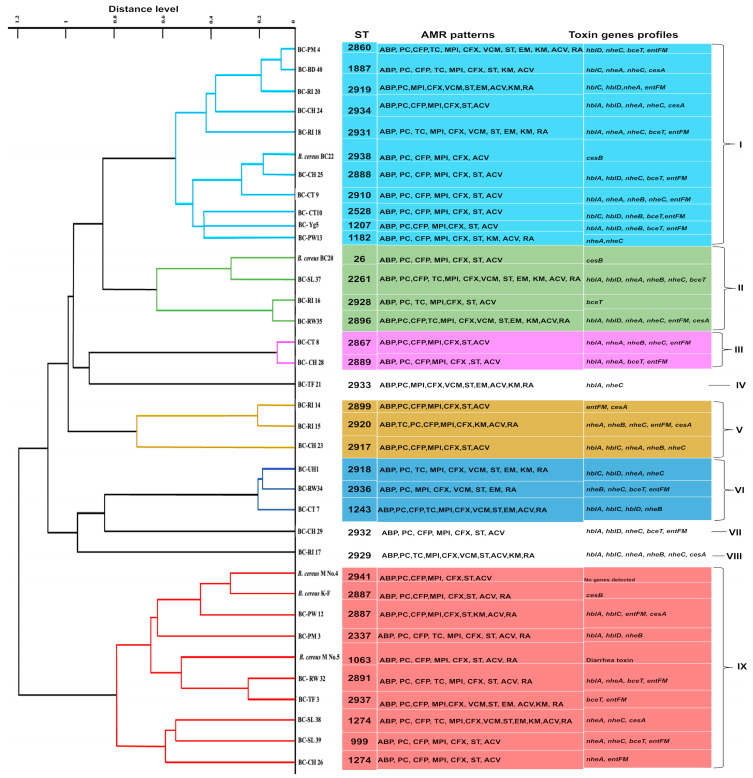
A principal component analysis (PCA) dendrogram of 36 *B. cereus s. s.* strains and their correlation with MDR patterns and enterotoxin profiles. MALDI-TOF MS data of the isolates were analyzed by PCA. Strains are grouped into nine clusters (numbered I–IX) based on MALDI-TOF MS results.

MALDI-TOF MS analysis of *B. cereus s. s.* isolates was performed, and a principal component analysis (PCA) dendrogram was generated using the Biotyper software version 4.0 to assess the similarity of the spectra among the tested isolates. The PCA dendrograms of the 36 different isolates are shown in Figure 1. The isolates were distributed into nine clusters (I–IX) on the PCA dendrogram. Some of the isolates that were assigned to the same cluster according to MALDI-TOF MS analysis had the same sequence type (ST), antimicrobial resistance patterns (AMR), sources, and biofilm-forming abilities, such as the *B. cereus* K-F and BC-PW12, and BC-SL 38 and BC-CH 26 isolates possessing the same ST (2887 and 1274, respectively). BC-SL39 and BC-26 isolates exhibited the same AMR pattern (ABP, PC, CFP, MPI, CFX, ST, and ACV) and were included in the same cluster IX. The BC-CH 24, BC-CH25, BC-CT9, and BC-Yg5 isolates that showed the same AMR patterns (ABP, PC, CFP, MPI, CFX, ST, and ACV) belonged to cluster I. Isolates BC-SL37 and BC-RW35 exhibited the same AMR patterns (ABP, PC, CFP, TC, MPI, CFX, VCM, ST, EM, KM, ACV, and RA) and were included in cluster II. Isolates BC-RI14 and BC-CH23 exhibited the same AMR patterns (ABP, PC, CFP, MPI, CFX, ST, and ACV), belonging to cluster V.

### 2.3. Virulence Gene Distribution among B. cereus s. s. Isolates

The *nheABC* gene cluster was present in 17.5% of the *B. cereus s. s.* isolates (Table 2 and Figure S1), with *nheA*, *nheB*, and *nheC* being found in 55, 32.5, and 62.5% of the isolates, respectively. The HBL genes *hblA*, *hblC*, and *hblD* were detected in 50, 30, and 42.5% of the isolates, respectively. In addition, 57.5% of the strains possessed the *entFM* gene. However, the *cytK* gene was not detected in any of the isolates, whereas the emetic toxin-synthetase *cesA* gene was detected in only seven strains (17.5%). Moreover, 35 different toxin gene distribution profiles were observed among the 40 isolates. The redundant genetic profiles present in the strains isolated from chicken meat showed that two isolates harbored the profile (*hblA*, *hblD*, *nheC*, *bceT*, and *entFM*), two isolates showed *(hblA*, *hblD*, *nheA*, *nheB*, and *nheC*) spectra, and two isolates bore only two genes (*nheA* and *entFM*). None of the isolates harbored any of the nine virulence genes, whereas one isolate harbored only one virulence gene (*bceT*).

### 2.4. Antimicrobial Susceptibility of B. cereus s. s. Isolates

**Figure 2:**
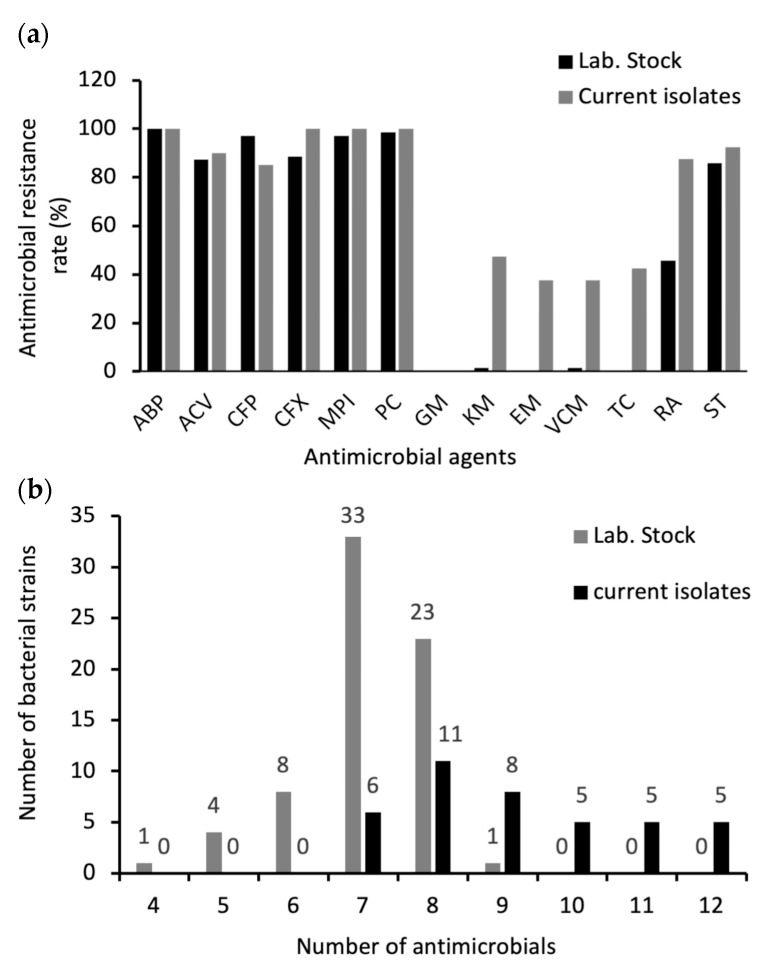
(**a**) Comparison between the rate of antimicrobial resistance of *B. cereus s. s.* isolated in different years to each antibiotic tested. (**b**) The multi-drug resistance pattern of *B. cereus s. s.* isolates (lab. stock and current isolates) in accordance with the number of antimicrobials used.

The antimicrobial resistance results for *B. cereus s. s.* isolates are presented in Table 3 and Figure 2a. Isolates (current and old laboratory stock) exhibited varying levels of resistance to different antibiotics, including β-lactams. However, all the current isolates were resistant to ampicillin, oxacillin, penicillin, and cefoxitin (100%), whereas 100%, 97.1%, 98.5%, and 88.5% of the laboratory stock strains were resistant to the same antibiotics, respectively. Most of the isolates were resistant to amoxicillin-clavulanic acid (90% and 87.1% for the current study and laboratory stock isolates, respectively). Moreover, 37.5% and 42.5% of 2022 isolates were resistant to erythromycin and tetracycline, respectively, whereas no resistance was observed against these two antimicrobials in laboratory stock strains. All *B. cereus s. s.* isolates were sensitive to gentamicin. Among the current isolates, 47.5% were kanamycin resistant, whereas only 1.4% of laboratory stock strains were found to be resistant to it. Notably, vancomycin resistance was detected in 15 isolates (37.5%) from newly isolated strains and only one strain (1.4%) from the laboratory stock. Figure 2b summarizes the multidrug resistance (MDR) profile of *B. cereus s. s.* isolates. In compliance with the definition of MDR [35], all *B. cereus s. s.* isolates were interpreted as MDR strains; however, the frequency of MDR was higher in the current strains than in the laboratory stock strains. All the isolates and 98.57% of the laboratory stock were resistant to seven or more antimicrobials. All the stock strains were resistant to five or more antimicrobials. ABP, PC, CFP, MPI, CFX, ST, ACV, and RA were the most common antibiotic-resistance patterns, with rates of 27.5% in current isolates and 32.8% in laboratory stock.

### 2.5. Distribution of Antimicrobial Resistance Genes in B. cereus s. s. Isolates 

The ratios of antimicrobial resistance of *B. cereus s. s.* isolates were high against tetracycline, erythromycin, and vancomycin. The presence of the corresponding resistance genes was detected using PCR. As presented in Table 4, 9 (52.9%) of the 17 tetracycline-resistant isolates in the current isolates were positive for tetracycline resistance gene (*tetA*) by PCR, and among the 15 erythromycin-resistant isolates, 12 (80%) were positive for the erythromycin resistance gene (*erm*). Of the 15 vancomycin-resistant isolates from the current isolates, 12, 3, 12, and 14 isolates were positive for *vanR*, *vanS*, *vanY*, and *vanW*, respectively. Other genes involved in vancomycin resistance (*vanA*, *vanB*, and *vanH*) were not detected in either laboratory stock or current isolates by PCR using the primers listed in Table S1.

### 2.6. Growth Profiles of B. cereus s. s. Isolates at 7 °C 

All tested strains were grown at 7 °C for day 7 (Table S2). However, the low-temperature growth profile varied among the *B. cereus s. s.* isolates. Twenty-five percent of the isolates grew rapidly and reached an optical density at 600 nm (OD_600_) of 0.8–0.9 value on day 7, whereas 20% of the isolates grew slowly and reached OD_600_ = 0.2–0.3 after the same incubation period. For the remaining strains, 22.50% and 32.5% reached OD_600_ = 0.4–0.5 and 0.6–0.7, respectively (Table 5).

### 2.7. Biofilm Formation of B. cereus s. s. Isolates

The biofilm-forming abilities of the 40 isolates were quantitatively assessed. All strains formed biofilms, albeit at different strengths, and were categorized into three groups. Seven (18%), nineteen (47.5%), and fourteen (35%) of the isolates showed weak, moderate, and strong biofilm formation, respectively. The specific biofilm formation (SBF) indices of each isolate and their categories are listed in Table 6. The sporulation rate within the attached biofilms varied from 0.001–0.5% of the total cells; strains that formed weak biofilms exhibited a rate of 0.002–0.003%, while strains that formed moderate or strong biofilms exhibited a rate of 0.01–0.5%.

### 2.8. Genetic Diversity of B. cereus s. s. Isolates

**Figure 3:**
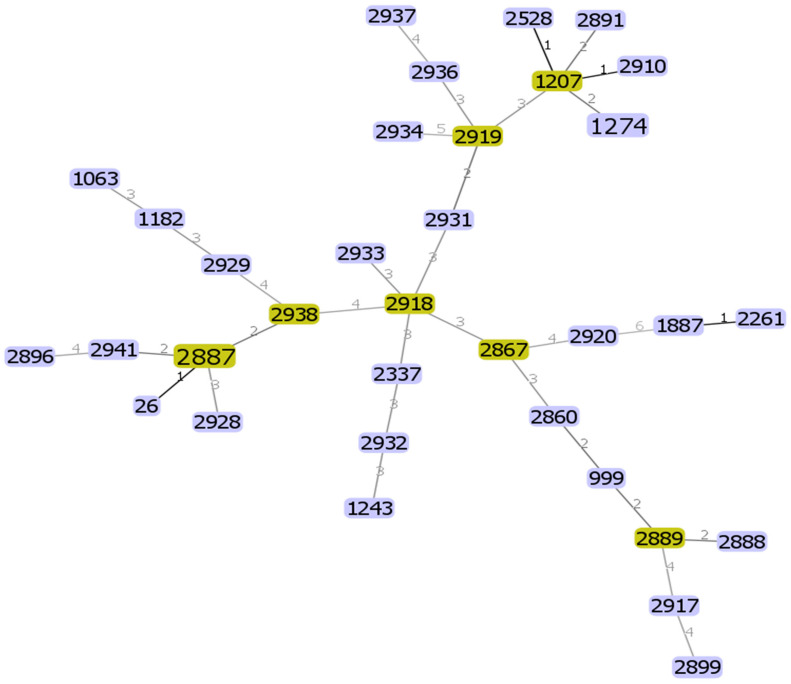
Genomic relationships between the 36 *B. cereus s. s.* isolates based on MLST data. The minimum spanning tree was constructed using the goeBURST algorithm implemented in the PHYLOViZ 2.0. The green color represents the subgroup founders (major nodes), while single-locus variants are highlighted with darker links. Rectangles: The sequence type of each isolate. Lines numbers: Number of different genes between isolates.

The genetic diversity of the 36 different *B. cereus s. s.* isolates was analyzed using the internal fragment sequences of seven housekeeping genes. In total, 34 different STs were identified (Table S3), 23 of which were novel, in addition to three new alleles, designated pur_387, tpi_344, and tpi_345, which were submitted to GenBank under the accession numbers OQ695781, OQ695782, and OQ695783, respectively. A minimal spanning tree was generated based on the sequences of seven housekeeping genes to estimate the relationships among the strains (Figure 3). All STs were represented by a single isolate except two STs represented by two isolates, each indicating a high level of genetic diversity among the *B. cereus s. s.* isolates. GoeBURST analysis revealed a close relationship between the yoghurt isolate ST1274 and four other STs (ST2528 and ST2910) isolated from cheese. These ST pairs are single-locus variants that differ from each other by a housekeeping gene allele. ST 1274 and ST 2891 were isolated from farm soil and raw milk, respectively, and are considered double-locus variants. Moreover, according to the PubMLST database, ST1207 from yoghurt and ST1243 from cheese in this study were previously isolated from fecal samples in South Korea; ST1274 in Japan; and ST2528 and ST2261 in China. ST1063 in this study from milk products was also previously isolated from milk products in China; ST1887 in North America; and ST 999 in many countries, including China, Korea, the Netherlands, and France. Our isolate, ST26, an emetic toxin-producing strain, was a widely recognized ST distributed worldwide and serves as a substantial ancestral clone of *B. cereus* [36]. Moreover, it has high pathogenic potential and is commonly encountered in clinical isolates [37]. Isolates ST2337 and ST2887 were identified in the PubMLST database as isolates from unknown sources. The other STs were novel and newly discovered in our isolates and pooled into the database. Of the twenty-three novel STs, nine isolates were from milk products, six from rice, five from chicken meat, two from food manufacturing facilities, and one from a tofu sample. These results suggest a diverse distribution of novel STs among different food sources, indicating the potential diversity of *B. cereus* strains in foods.

## 3. Discussion

*B. cereus s. s.* is a common foodborne pathogen associated with gastrointestinal disorders. In addition, it can grow well at room temperature in starchy foods to produce emetic toxins. Emetic and diarrheal syndromes caused by *B. cereus s. s.* are associated with different food types due to the specific toxins involved and their production environments. Emetic syndrome is commonly linked to starch-rich foods like rice and pasta because the emetic toxin, cereulide, is produced in these foods before ingestion and remains active even after cooking. In contrast, diarrheal syndrome is associated with protein-rich foods such as meat, dairy, and vegetables, where enterotoxins (NHE, HBL, and cytotoxin K) are produced in the host’s intestine, favored by the nutrient-rich environment provided by these foods. These differences in food association are due to the distinct metabolic pathways and environmental conditions that favor the production of each type of toxin by *B. cereus s. s.* strains [37,38]. 

Some cases of food poisoning have been reported due to the consumption of cooked rice, noodles, or sticky rice cakes [39], especially in Japan [40]. In December 2001, in Kumamoto Prefecture, Japan, 346 people experienced food poisoning caused by emetic toxins after eating sweet red bean paste with sticky rice cake (*An-iri-mochi*) [39,41,42]. Despite causing many foodborne illnesses annually, *B. cereus* outbreaks have rarely been investigated or reported. 

This study investigated the prevalence and characteristics of *B. cereus s. s*. strains isolated from various food and environmental samples. The isolates were confirmed to be *B. cereus s. s.* via morphological, biochemical, and genetic analyses. The findings revealed that the contamination rate with *B. cereus s. s.* was 22.34% in the samples tested, highlighting its presence in various food products. According to previous studies, the contamination rate of *B. cereus* in various types of food ranges from 6.8% to 57% [34]. In Brazil, out of 180 UHT milk samples, 25 (13.8%) were *B. cereus*-positive [43]. Furthermore, 27.2% of raw milk produced in the Czech Republic [44] and 21% of meat over rice dishes and miscellaneous foods in Thailand [45] has also tested positive. *B. cereus* was isolated from different starchy foods in Japan, and 2 of the 10 isolates were from processed rice products [46]. Closely related isolates were differentiated by combining MALDI-TOF MS analysis with PCA. PCA offers a quick qualitative method to assess the relationships among the isolates and evaluate large datasets, such as protein profiles [47]. The dendrogram generated by PCA analysis based on the MALDI-TOF MS data revealed nine clusters, suggesting high protein diversity among the isolates. Some isolates derived from the same source were classified into the same cluster, which is consistent with previous reports [48]. Additionally, some isolates with identical antibiotic resistance patterns and biofilm-forming abilities were grouped together, as in cluster VI, and the three strains included in this cluster showed vancomycin resistance. However, considerable variation was observed among the isolates in the same cluster regarding antimicrobial resistance and ST. Even within the same species or cluster, bacterial populations can exhibit substantial genetic diversity. Horizontal gene transfer, mutations, and genetic recombination contribute to differences in antimicrobial resistance genes and other genetic elements, leading to distinct resistance profiles among isolates [49]. PCA analysis of the isolates demonstrated effective classification of the isolates at the species level through cluster formation in the proteomic dendrogram generated from Bruker Biotyper MALDI-TOF MS (Bruker Daltonik GmbH, Bremen, Germany) spectra. Previous research has shown that combining MALDI-TOF MS with PCA is a powerful method for the rapid identification and classification of *Virgibacillus* and *Bacillus* strains isolated from the same species [47].

Numerous studies have confirmed the presence of virulent *B. cereus s. s.* in various food products. This study identified various toxigenic genes linked to *B. cereus s. s.* virulence among the isolates. All the isolates possessed genes for at least one toxin, although none of the isolates carried the *cytK* gene [50]. The *nheABC* gene cluster was present in 17.5% of the *B. cereus* isolates, which was lower than that in *B. cereus* strains from different food samples and clinical isolates associated with foodborne outbreaks in previous studies [51,52,53]. In addition, 57.5% of the strains possessed the *entFM* gene. The most frequently identified enterotoxin genes in *B. cereus s. s.* strains were *nhe* and *entFM*, consistent with previous findings [30,54,55,56]. The percentage of NHE-producing strains was greater than that of HBL-producing strains across all sample types [57]. The emetic toxin-synthetase gene *cesA* was present in only seven strains (17.5%), which was lower than that in a previous study [58], which detected the *ces* gene in 25 of 35 Korean fermented soybean paste isolates (71%). However, it was similar to other recent studies (9%, [59]; 10.2%, [60]), and higher than that of another study [32] (5%). Although *B. cereus s. s.* is prevalent in various foods and environments, the occurrence of emetic strains carrying the *ces* gene is generally low. The varied prevalence of toxigenic genes indicates that virulence factors can differ significantly depending on the environment and geographic location, impacting the severity and type of illness caused by *B. cereus s. s.* strains [61].

Antibiotic susceptibility testing showed that MDR was higher in the current *B. cereus s. s.* strains than strains that were isolated 20 years ago. Most of the isolates, both current and the older laboratory stocks, were resistant to β-lactam antibiotics due to their natural production of β-lactamase enzymes that hydrolyze the β-lactam ring of antibiotics, rendering them ineffective. This allows *B. cereus* to survive in environments where these antibiotics are present [62]. These results are similar to those of previous studies [58,63,64]. Notably, resistance to tetracycline, erythromycin, and vancomycin was not detected in the old laboratory stock strains, except for one vancomycin-resistant strain. In the new isolates, the resistance to vancomycin and erythromycin increased to 37.5% of the isolates, while tetracycline resistance was found in 42.5% of the isolates. Our findings are consistent with those of a previous study [65], which reported high resistance to vancomycin (63%), tetracycline (86%), and erythromycin (42%). Similarly, a previous study [66] also showed the resistance of isolates to vancomycin (17.6%), tetracycline (26.5%), and erythromycin (50%). Additionally, *B. cereus* isolates exhibited resistance to vancomycin, tetracycline, and erythromycin, with three, two, and five isolates, respectively, in [67]. Resistance to vancomycin, tetracycline, and erythromycin was also detected in 20.24%, 17.86, and 15.47% of the isolates, respectively, in [57]. In addition, a previous study found that 13% of *Bacillus* isolates were resistant to vancomycin [32]. Some *B. cereus s. s*. strains have developed resistance to vancomycin through a combination of phenotypic and genotypic mechanisms. This resistance may be attributed to modifications in cell wall peptidoglycan precursors, specifically the replacement of D-alanyl-D-alanine with D-alanyl-D-lactate, which impairs vancomycin’s ability to inhibit cell wall synthesis. Additionally, the presence of efflux pumps and the acquisition of new resistance genes may also contribute to the *B. cereus* resistance [57]. All of our isolates were sensitive to gentamicin, consistent with the results of a previous study [68]. Vancomycin is considered one of the most appropriate choices for treating *B. cereus* infections [32]. Several studies have demonstrated its efficacy in managing these infections, and one study [6] has indicated that vancomycin is a reliable choice for empirical therapy in *B. cereus* bloodstream infections, confirming its suitability in clinical settings. The combination therapy of vancomycin and gentamicin was effective in treating persistent *B. cereus* bacteremia in a 56-year-old woman [69]. Another study [70] demonstrated that oral vancomycin is an appropriate treatment for managing food poisoning, focusing on a case involving two sisters who had consumed pasta that had been cooked three days earlier resulting in severe illness caused by a cereulide-producing strain of *Bacillus cereus*, which led to multi-organ dysfunction syndrome due to the preformed emetic toxin. Despite this, some of our isolates, in addition to those reported in previous studies, showed resistance to vancomycin. One study examined a case of a previously healthy young man who had developed vancomycin-resistant *B. cereus* pneumonia and bacteremia and died of the complications of this disease. No improvement was seen in the patient after treatment with vancomycin, raising concerns for antibiotic resistance and the potential virulence of *B. cereus* [71]. This indicated the emergence of vancomycin-resistant *B. cereus* infections over time, highlighting that reliance on vancomycin may not be effective in all cases and underscoring the need for alternative treatment options other than antibiotics to ensure the successful management of these infections [72].

Despite numerous phenotypic antimicrobial susceptibility studies on *B. cereus s. s.* in food products, there is a lack of data on the prevalence of antimicrobial resistance genes (ARGs) in *B. cereus s. s.* that persist in food. This study examined the genotypic profile of resistance of *B. cereus s. s* isolates against tetracycline, erythromycin, and vancomycin. The predominant mechanism of vancomycin resistance in phenotypically resistant strains is mediated by *vanW* and *vanY*, which are responsible for peptidase activity and bacterial defense against vancomycin, respectively. The *tetA* gene is responsible for tetracycline resistance in 52% of phenotypically resistant strains [73], and *tetA* is the primary gene encoding resistance in *B. cereus s. s.* isolates. Other studies in Japan detected *tet*A and *tet*B genes in *B. cereus s. s.* isolates from Japanese black beef cattle [74]. However, none of these genes involved in the phenotypic antibiotic resistance were detected by PCR in the isolates. Phenotypic and genetic profiles of antimicrobial resistance of bacteria do not always match those of a given antimicrobial. Several factors may explain this discrepancy; gene mutations or other mechanisms may contribute to antimicrobial resistance in *B. cereus* [74]. In Japan, more than half of the antimicrobials used in livestock production are veterinary antimicrobials and feed additives [75]. The extensive use of antimicrobials in livestock can substantially affect livestock products, especially milk and dairy products. Residues of these antimicrobials can be transferred into milk, potentially leading to antibiotic contamination that poses health risks to consumers [76]. Studies have demonstrated a link between the extensive use of veterinary antimicrobials and the increased antimicrobial resistance of bacteria (AMR) isolated from food-producing animals. It has been reported that the AMR strain exhibited resistance to commonly used antimicrobials [77]. This rise in AMR strains, driven by antimicrobial usage, is very important because of the extensive usage of antimicrobials and antimicrobial feed additives in Japan. The Japanese Veterinary Antimicrobial Resistance Monitoring System (JVARM) has indicated that the emergence and spread of resistant *Escherichia coli* to many antibiotics including tetracycline were likely associated with the therapeutic use of antimicrobials in food-producing animals. The use of antimicrobials in veterinary medicine influences the development, spread, and prevalence of antimicrobial resistance in bacteria isolated from food-producing animals [78]. Annual reports indicate that tetracyclines have been used in the highest quantities in Japan [79]. More antibiotics are used on farms than that used in humans. The annual amount of antibiotics used in Japan is 581.3 tons/year for humans and 915.5 tons/year for livestock animals, including feed additives. Therefore, livestock farms are considered an important source of antibiotic-resistant bacteria, which may even be transmitted to humans via meat and milk [77,80,81]. The presence of antibiotic-resistant *B. cereus s. s.*, particularly MDR strains, in food products highlights the role of food systems as reservoirs of ARGs.

All the isolates in the present study were psychrotrophic, such as those found in refrigerators. This phenomenon promotes secondary contamination by *B. cereus s. s.* during refrigerated storage, potentially causing the spread of spoilage between refrigerated foods. Similarly, according to a previous study [52], nearly all the involved *B. cereus* isolates (91.3%) were psychrotrophic. Another study reported that 25% of their isolates could grow at 7 °C [2].

Biofilm formation in the food industry has substantial medical and economic implications. However, it is uncertain whether biofilms enhance *B. cereus s. s.* sporulation compared to planktonic growth. *B. cereus s. s.* biofilms mainly consist of vegetative cells; however, spores can develop within these biofilms during maturation and aging. Previous studies have indicated that spores in biofilms may comprise 0.01–10% of total cells, varying among strains [82]. In our study, all the isolates produced biofilms to different extents, and the sporulation rate was low. Further studies are required to clarify the risk of sporulation by *B. cereus s. s.* in the biofilms. Psychrotrophic *B. cereus* is a significant concern in the dairy industry due to its ability to form biofilms and proliferate at low temperatures, leading to persistent contamination, food spoilage, and potential health risks. As a result, *B. cereus s. s.* is considered a major reason for dairy product spoilage and economic losses in the industry. The persistence of biofilms on industrial equipment results in both pre- and post-processing contamination, which in turn reduces the shelf life of products [83,84].

MLST is a vital epidemiological typing technique commonly employed in studies exploring the evolution and population diversity of *B. cereus s. s.* isolates [85]. Based on the MLST profiles, the current isolates exhibited genetic diversity. Among the 36 *B. cereus s. s.* isolates, 23 were novel. Similarly, other studies have found high genetic diversity among isolated *B. cereus s. s.* strains [53,86,87,88]. However, no correlation was found between ST profiles and the prevalence of virulence genes, and each ST exhibited a unique combination of virulence genes, indicating substantial differences between the isolates. This study highlighted the high level of genetic diversity among *B. cereus s. s.* isolates, which is consistent with their varied virulence gene profiles and MALDI-TOF MS patterns. This diversity refers to the evolution and emergence of potentially more virulent and antimicrobial-resistant bacteria, which underscores the complex pathogenic potential of *B. cereus s. s*. Further studies should be performed on *B. cereus s. s*. strains isolated from a wide range of samples from various regions all over the world to expand the MALDI-TOF MS spectra library for a better understanding of the epidemiology, pathogenicity, and antibiotic resistance mechanisms of *Bacillus* species. The results raise concern about the status of the emergence of multidrug-resistant *B. cereus s. s.*, which requires conducting more extensive studies across Japan to evaluate *B. cereus s. s.* contamination levels using a larger sample size and enhanced resources. This will ensure a more comprehensive and accurate assessment of its prevalence and impact. The specific genes involved in biofilm formation will be determined for the isolates and a detailed analysis of these genes and their functional roles, as well as their contributions to virulence and resistance characteristics, will be performed in future research.

## 4. Materials and Methods

### 4.1. Sample Collection 

A total of 179 samples were collected from different local markets and farms in the area around the Ito campus of Kyushu University in Fukuoka, Japan. This included food samples, consisting of 50 raw chicken meat samples (breast, minced, chopped meat, wing, thigh, and skin), 89 milk and milk product samples (raw milk, pasteurized milk, ultra heat-treated milk (UHT), ice cream, cheese, yoghurt, and milk protein), five tofu samples, and 20 ready-to-eat cooked rice samples. Additionally, fifteen soil and bedding samples were collected from cow-grazing areas. The sampling strategy was designed to include a wide range of commonly consumed food products, along with relevant environmental samples, covering most of the known vehicles for *B. cereus* contamination. Samples were collected randomly from different supermarkets and farms in Fukuoka and different brands with an average of four samples/supermarket from December to April. The number of samples in each category was determined based on prevalence data from previous studies, sample availability, and the need to ensure statistical relevance. All samples were transported to the laboratory at 4 °C within 3 h and bacteriological examinations were promptly performed. Sixty-nine strains of *B. cereus s. s* (including diarrhea and emetic toxin-producing strains) isolated from dairy products and the processing lines of milk-processing factories in 2004) were also used to compare antibiotic resistance between the *B. cereus s. s.* strains isolated in this study. These strains were characterized and biochemically identified as *B. cereus s. s.* and stored at −80 °C in 20% glycerol [89]. In addition, *B. cereus* JCM 21512 was used as a positive control for testing by polymerase chain reaction (PCR).

### 4.2. Isolation and Identification of Bacterial Strains

Sample preparation and isolation of *B. cereus s. s* were performed in accordance with the National Food Safety Standards of China (GB4789, 14-2014) [90]. Briefly, 25 mL/g of each sample was mixed with 225 mL of Tryptic Soy Broth (TSB) (Becton, Dickinson, and Co., Franklin Lakes, NJ, USA), homogenized, and incubated at 37 °C for 24 h. A loopful of the incubated sample was then spread onto Compact Dry^a^ X-BC agar plates (CD-XBC; Nissui Pharmaceutical Co., Ltd., Tokyo, Japan) and incubated at 37 °C for 24 h. Green-blue colonies were purified on the same medium and stored at −80 °C in 20% glycerol. All frozen isolates were refreshed in TSB, incubated at 37 °C for 24 h, sub-cultured on chromogenic media BACARA (Biomerieux, Hampshire, UK), and incubated at 30 °C for 24 h. Pink-orange colonies with a precipitate zone indicating lecithinase production were selected for further identification and confirmation of *B. cereus s. s.* and differentiation between other *B. cereus* group members, according to the FDA’s Bacteriological Analytical Manual [91]. 

### 4.3. MALDI-TOF MS Analysis of B. cereus s. s. Isolates

*B. cereus s. s.* isolates were subjected to Biotyper MALDI-TOF MS (Bruker Daltonik GmbH., Bremen, Germany) for identification. This method uses proteomic analysis to generate spectral profiles of the tested isolates, which are then compared to a library of well-characterized microorganisms. Briefly, specimens were cultured on tryptic soy agar (TSA) at 37 °C for 24 h. Subsequently, single colonies were suspended in 300 μL distilled water, vortexed for 30 s, and treated with 99.5% ethanol (900 μL). The samples were stored at −20 °C until MALDI-TOF MS analysis was performed at Fukuoka Sangyo University, Fukuoka, Japan. MALDI-TOF MS analysis was performed in triplicate for each isolate. A PCA dendrogram was generated using the Biotyper software version 4.0 to assess protein similarity among the tested isolates [48]. Proteins were extracted from the bacterial isolates and analyzed using MALDI-TOF MS. Proteins having a *m*/*z* in the range between 2000 and 20,000 *m*/*z* were used for the identification of bacterial strains based on individual mass peaks corresponding to specific ribosomal proteins. The mass spectra obtained were preprocessed with the Biotyper software version 4.0, including baseline subtraction, normalization, and peak detection. PCA is a statistical technique used to reduce the dimensionality of the dataset and visualize protein similarity. This method analyzes matrices of variance-covariance and correlations of data. Peaks obtained from MALDI-TOF, potentially representing proteins or peptides, were used for clustering spectra with similar characteristics. The data can be represented in either a 2D or a 3D coordinate system. The analyses, including PCA and dendrograms, followed standard procedures using built-in software. Raw spectra from triplicates were preprocessed with Flex Analysis 3.4 software, involving smoothing and baseline subtraction, to ensure quality. The distance between the clusters showed the variation at the group level, while the distance between the strains (within the cluster) showed the differences in protein profiles at the strain level [47,92].

### 4.4. Molecular Characterization and Toxin Gene Profiling of B. cereus s. s. 

PCR identification was conducted targeting the *gyrB* gene using the primer pair BC1/BC2r for *Bacillus cereus s. s.* and the primer pair BT1/BT2r for *B. thuringiensis* [93]. *B. cereus* JCM2152, obtained from the Japan Collection of Microorganisms (Wako, Saitama, Japan), and *B. thuringiensis* IFO 3951, obtained from the Institute for Fermentation, Osaka, were used as reference control strains. Furthermore, the presence of nine enterotoxigenic genes (*hblA*, *hblC*, *hblD*, *nheA*, *nheB*, *nheC*, *cytK*, *bceT*, and *entFM*) and one cereulide synthetase gene (*ces*) was detected by colony PCR. For the *ces* gene, a pair of *CesF1*/*CesR2* was employed to target the *cesB* gene [94], in addition to a new primer designed in our laboratory that targeted the *cesA* gene for further confirmation of its presence; this was performed to specifically and effectively target highly functional domains of the known non-ribosomal peptide synthetase (cereulide), as described by [95]. Three cereulide-positive strains from our laboratory stock were used as the controls [89]. The primers, annealing temperatures, and amplicon sizes used in this study are listed in Table S4. *B. cereus* colonies were randomly picked up from the TSA plates and suspended in 30 μL of pure autoclaved water. The suspension was used as the template DNA for colony PCR. The PCR reaction mixture (10 μL) comprised 5 μL of 2 × GoTaq^®^ Green Master Mix (Promega Corp., Madison, WI, USA), 0.5µM of each primer solution (concentration 10 μM each), 1 µL of template DNA, and nuclease-free water (3 μL). A DNA template containing a single colony was cultured on TSA and dissolved in the PCR mixture. PCR was performed using Takara PCR Thermal Cycler Dice (Takara Shuzo Co., Tokyo, Japan). The amplicons were analyzed using gel electrophoresis on a 1.5% agarose gel with Midori Green Advance DNA stain (Nippon Genetics Co., Ltd., Tokyo, Japan) at 100 V for 25 min, and visualized using LuminoGraph I (ATTO, Tokyo, Japan). A 100 bp + 3 K DNA ladder (SMOBIO, Inc., Hsinchu City, Taiwan) was used as a standard to measure the PCR product sizes.

### 4.5. Antimicrobial Resistance Testing

#### 4.5.1. Disk Diffusion Method

The phenotypic antibiotic resistance of *B. cereus s. s.* isolates was screened using the Kirby–Bauer disk diffusion assay according to the M100 Clinical and Laboratory Standards Institute guidelines [96]. The forty isolated strains from the current study, in addition to 69 from the laboratory stock that were isolated and characterized fully in our laboratory 20 years ago [89], and *B. cereus* JCM 21512 were subjected to antimicrobial resistance testing, A total of 13 antimicrobial disks were used (Eiken Chemicals, Tokyo, Japan): ABP, 10 µg; TC, 30 µg; PC, 10 U; CFP, 30 µg; MPI, 1 µg; CFX, 30 µg; VCM, 30 µg; GM, 10 µg; ST, 1.25–23.75 µg; EM, 15 µg; KM, 30 µg; ACV, 20/10 µg; and RA, 5 µg. Bacterial suspensions in saline were adjusted to 0.5 McFarland turbidity of 600 nm and streaked onto Müller Hinton agar (Becton Dickinson and Company). Antibiotic disks were added, and the plates were incubated overnight at 37 °C. After incubation, the plates were examined for zones of inhibition around the disks, where bacterial growth had been prevented. The susceptibility of the isolates to the antibiotics was determined based on the diameter of the inhibition zone. The diameter of these inhibition zones was measured in millimeters and then was interpreted as sensitive, intermediate, or resistant according to CLSI [96], the interpretative standard zone diameters of the different antimicrobials used were presented in Table S5. *Staphylococcus aureus* JCM 2413, derived from ATCC 25923, was used as a quality control.

#### 4.5.2. Molecular Identification of Genes Involved in Antibiotic Resistance

Based on the phenotypic antimicrobial resistance patterns, resistant isolates were selected and cultured overnight in TSB at 37 °C. Genomic DNA was extracted from *B. cereus s. s.* isolates and subjected to PCR detection of the ARGs *erm*; *tetA*; and *van A*, *B*, *R*, *S*, *Y*, *W*, and *H*. Based on the phenotypic antimicrobial resistance patterns, resistant isolates were selected and cultured overnight in TSB at 37 °C. Genomic DNA was extracted for PCR detection of ARGs. Primer sets for the detection of *van R*, *van S*, *van Y*, *van W*, and *van H* were specifically designed. Novel primers were designed using the Primer 3 input (version 0.4.0) based on homologous bacterial sequences. Reference sequences for ARGs were obtained from the NCBI for Biotechnology Information (www.ncbi.nlm.nih.gov, accessed on 15 November 2022). The whole-genome sequence of the vancomycin-resistant reference strain *B. cereus* ATTC 14579, sourced from the KEGG PATHWAY database (https://www.genome.jp/kegg/pathway.html, accessed on 1 December 2022), was used to localize the vancomycin-resistance gene cluster. The antimicrobial resistance oligonucleotide sequences are listed in the Supplementary Material (Table S1). A genomic DNA solution (1 of 50 ng DNA) was used in a 10 μL reaction mixture consisting of 5 μL of 2 × GoTaq^®^ Green Master Mix (Promega Corp., Madison, WI, USA), 0.5 μL of 1 μM each of the primers, and nuclease-free water (3 μL). PCR conditions were as follows: pre-heating at 94 °C for 5 min, 30 PCR cycles of denaturation at 98 °C for 10 s, annealing for 30 s, and elongation at 72 °C for 1 min, followed by final elongation at 72 °C for 5 min. The annealing temperatures for each primer are listed in Table S1. PCR products were subjected to 1.5% agarose gel electrophoresis stained with Midori Green Advance DNA stain (Nippon Genetics) and visualized on WSE-6100H LuminoGraph I (ATTO).

### 4.6. Discrimination of Psychrotrophic Strains of B. cereus s. s. 

*B. cereus s. s.* strains were grown at 37 °C on TSA plates. A single colony was selected and suspended in TSB tubes at 37 °C for 18 h. Ten microliters of the solution from the incubated tube was transferred to a new tube, and the initial OD value was measured. The tubes were incubated at 7 °C for 7 days. OD values were measured on days 3, 5, and 7. The experiments were repeated thrice on separate days.

### 4.7. Biofilm Formation Assay

The biofilm-forming ability of *B. cereus s. s.* isolates was detected using the microtiter plate biofilm formation method as previously described [97,98] with minimal changes. Briefly, bacterial isolates were grown overnight to the stationary phase in BHI broth, as it is the optimum medium for supporting biofilm formation but does not support spore formation [99,100]. Ninety-six-well plates filled with 200 μL BHI were inoculated with 10 μL of overnight suspension and incubated at 37 °C for 24 h. The broth was replaced with fresh BHI medium and incubated for 24 h. The OD was determined at 600 nm. Non-adherent planktonic cells were removed, and the broth was collected for sporulation rate detection and microscopic examination. One plate was used as a control. Wells were washed with phosphate-buffered saline (PBS), air-dried, and stained with 2 mL of 0.3% crystal violet for 5 min. After washing with distilled water, the stained biofilms were solubilized in 70% ethanol, and the OD was measured at 540 nm. Biofilm quantity was estimated using the specific biofilm formation (SBF) index (AB-CW)/G. The assay was performed in triplicates and mean values were considered for interpretation. Isolates were classified as strong (SBF > 1), moderate (SBF = 0.5–1), or weak (SBF < 1). This experiment was repeated thrice. *B. cereus s. s.* biofilms mainly consist of vegetative cells that produce spores during maturation [96,99]. To determine the sporulation rate, the sporulation efficiency in the biofilms was measured by comparing the number of spores after heating with the total cell count before heating [98]. After 48 h of incubation, the bacterial suspension was heated to 80 °C for 10 min, cooled, and centrifuged. The pellet was resuspended in PBS, serially diluted, and spotted onto BHI agar plates for overnight incubation at 30 °C. The control plates were serially diluted and incubated. Spore numbers were determined using plate counting of the CFU.

### 4.8. Multi-Locus Sequence Typing

#### 4.8.1. DNA Extraction

*B. cereus s. s.* isolates were grown at 37 °C overnight in TSB. DNA was extracted using a Cica Geneus DNA Extraction Kit (Kanto Chemical Co., Inc., Tokyo, Japan) according to the manufacturer’s instructions. The quality of extracted DNA was confirmed using a Nanodrop ND-1000 spectrophotometer (Nanodrop Technologies, Wilmington, DE, USA). The extracted DNA was stored at −20 °C in an elution buffer until use.

#### 4.8.2. Genes Amplification, Sequencing, and Determination

The internal fragments of seven housekeeping genes (*glp*, *gmk*, *ilvD*, *pta*, *pur*, *pycA*, and *tpi*) were amplified using PCR (Takara Shuzo Co.) from the extracted bacterial DNA using specific primers and annealing temperatures following the MLST protocol for *B. cereus* in the PubMLST database (https://pubmlst.org/organisms/bacillus-cereus/primers, accessed on 15 August 2022). PCR was performed using EX Taq DNA polymerase (Takara Bio Inc., Kusatsu City, Japan). The PCR amplification program included: initial denaturation at 95 °C for 3 min, followed by 30 cycles of denaturation at 94 °C for 30 s, annealing at 55–59 °C depending on the primer used for 60 s, extension at 72 °C for 30 s, and a final extension at 72 °C for 7 min [101]. The PCR products were purified using the FastGene^®^ PCR/Gel Extraction Kits (Nippon Genetics) and then sequenced using Sanger DNA sequencing (Genewiz Inc., Tokyo, Japan). Nucleotide sequences were pooled into the PubMLST database to obtain the corresponding allele numbers. If no exact match was observed, a new allele was assigned. The sequence of each strain was determined. A novel ST number was assigned if there was a new allelic combination and the novel STs obtained were submitted to the current database. The completed sequences were compared with those in the *B. cereus* MLST database. All new sequences were submitted to the database, and relevant data are available at http://pubmlst.org/bcereus (accessed on 1 January 2023). Further Burst Analysis was performed on all obtained STs using PHYLOViZ 2.0 (Instituto de Microbiologia, Lisboa, Portugal) to divide strains into groups according to their allelic profiles [33,102].

### 4.9. Statistical Analysis

The virulence gene distribution, biofilm formation, and antimicrobial resistance were performed in triplicate The data were analyzed as % values, means, and standard deviations using descriptive statistics (Microsoft Excel^TM^ 2024).

## 5. Conclusions

This is the first comprehensive study on the prevalence of *B. cereus s. s.* in various foods in Fukuoka, Japan. These findings indicate a potential risk because the isolates were multi-toxigenic and multidrug-resistant, especially vancomycin-resistant, which complicates the treatment of *B. cereus s. s*. infections. The drastic increase in antimicrobial resistance, particularly to vancomycin and tetracycline, highlights a growing problem that poses a substantial global threat. MALDI-TOF MS and MLST analyses revealed significant differences among the strains, with extensive diversity at both the protein and genomic levels. The combination of these methodologies highlights the considerable heterogeneity of *B. cereus s. s.* isolates, reflecting high diversity in both proteomes and genomes. Some isolates formed robust biofilms and grew at low temperatures, suggesting an increased threat to food safety. These results underscore the need for stringent measures to manage *B. cereus s. s*. contamination in the food industry. Furthermore, new genes and novel STs were identified, which will enrich existing databases and substantially contribute to the scientific community. Future research should focus on finding natural alternatives to antibiotics for the biocontrol of *B. cereus s. s.* to overcome the antibiotic resistance issue.

## Figures and Tables

**Table 1:** Occurrence of *B. cereus s. s.* in various food and soil samples tested.

Tested Sample	No. of Positive Samples by the Method (%)			Total Samples Collected
	XBC	BACARA	PCR	
Milk and milk products	40	22	18	89
Raw chicken meat	21	9	9	50
Tofu	3	3	3	5
RTE cooked rice	11	7	6	20
Soil and bedding	13	8	4	15
Total No. (%)	88 (49.1%)	50 (27.3%)	40 (22.3%)	179

**Table 2:** Prevalence of the virulence genes among *B. cereus s. s.* isolates.

Virulence Genes	Number of Isolates (%) Positive for Target Gene (s)
NHE gene complexes	
*nheA*	22 (55%)
*nheB*	13 (32.5%)
*nheC*	25 (62.5%)
*nheA + nheB + nheC*	7 (17.5%)
HBl gene complexes	
*hblA*	20 (50%)
*hblC*	12 (30%)
*hblD*	17 (42.5%)
*hblA + hblC + hblD*	1 (2.5%)
*bceT*	17 (42.5%)
*entFM*	23(57.5%)
*Ces A*	7 (17.5%)
*cytK*	0 (not detected)

**Table 3:** Antibiogram pattern of *B. cereus s. s.* isolates.

Category	Antimicrobial Agents	*B. cereus s. s.* Strains No. (%)					
		Current Isolates (*n* = 40)			Lab. Stock (*n* = 70)		
		Resistance	Intermediate	Susceptible	Resistance	Intermediate	Susceptible
β lactam Antibiotics	Ampicillin (ABP) 10 µg	40 (100%)	0 (0)	0 (0)	70 (100%)	0 (0)	0 (0)
	Amoxicillin-Clavulanic acid (ACV) 20 µg/10 µg	36 (90%)	0 (0)	4 (10%)	61 (87.1%)	0 (0)	9 (12.8%)
	Cefepime (CFP) 30 µg	34 (85%)	6 (15%)	0 (0)	68 (97.1%)	2 (2.8%)	0 (0)
	Cefoxitin (CFX) 30 µg	40 (100%)	0 (0)	0 (0)	62 (88.5%)	0 (0)	8 (11.4%)
	Oxacillin (MPI) 1 µg	40 (100%)	0 (0)	0 (0)	68 (97.1%)	1 (1.4%)	1 (1.42%)
	Penicillin (PC) 10 units	40 (100%)	0 (0)	0 (0)	69 (98.5%)	0 (0)	1 (1.42%)
Aminoglycosides	Gentamicin (GM) 10 µg	0 (0)	0 (0)	40 (100%)	0 (0)	0 (0)	70 (100%)
	Kanamycin (KM) 30 µg	19 (47.5%)	5 (12.5%)	16 (40%)	1 (1.4%)	2 (2.8%)	67 (95.7%)
Macrolides	Erythromycin (EM) 15 µg	15 (37.5%)	1 (2.5%)	24 (60%)	0 (0)	11 (15.7%)	59(84.2%)
Glycopeptides	Vancomycin (VCM) 30 µg	15 (37.5%)	1 (2.5%)	24 (60%)	1 (1.4%)	0 (0)	69 (98.5%)
Tetracyclines	Tetracycline (TC) 30 µg	17 (42.5%)	5 (12.5%)	18 (45%)	0 (0)	2 (2.8%)	68 (97.1%)
Rifamycin	Rifampicin (RA) 5 µg	35 (87.5%)	2 (5%)	3 (7.5%)	32 (45.7%)	26 (37.1%)	12 (17.1%)
Folic acid Inhibitors	Trimethoprim-Sulfamethoxazole (ST) 1.25 µg–23.75 µg	37 (92.5%)	3 (7.5%)	0 (0)	60 (85.7%)	7 (10%)	3 (4.2%)

**Table 4:** Proportions of antimicrobial resistance genes in tested antibiotic-resistant *B. cereus s. s.* isolates.

Isolates	Total No.	No. of Positive Isolates (% in Resistant Isolates)											
		Tetracycline		Erythromycin		Vancomycin							
		Resistant Strain No.	*tetA*	Resistant Strain No.	*erm*	Resistant Strain No.	*vanA*	*vanB*	*vanR*	*vanS*	*vanY*	*vanW*	*vanH*
Lab. stock	70	0	0	0	0	1	0	0	1 (100)	1 (100)	1 (100)	1 (100)	0
Current	40	17	9 (52.9)	15	12 (80)	15	0	0	12 (80)	3 (20)	12(80)	14 (93.3)	0

**Table 5:** Growth of *B. cereus s. s.* isolates at 7 °C for 7 days.

	OD_600_ at 7th Day				Total
	0.2–0.3	0.4–0.5	0.6–0.7	0.8–0.9	
No. of strains grown	8	13	9	10	40
Percentage	20%	32.5%	22.5%	25%	100%

**Table 6:** Biofilm formation ability of *B. cereus s. s.* isolates.

Strain Code	SBF Index ± SD	Biofilm Forming Strength	Strain Code	SBF Index ± SD	Biofilm Forming Strength
BC-UH 1	0.31 ± 0.02	weak	BC-TF 21	1.59 ± 0.10	strong
BC-UH 2	0.26 ± 0.04	weak	BC-TF 3	1.16 ± 0.07	strong
BC-PM 3	0.89 ± 0.01	moderate	BC-CH 23	1.75 ± 0.04	strong
BC-PM 4	0.52 ± 0.02	moderate	BC-CH 24	0.45 ± 0.02	weak
BC-Yg5	0.61 ± 0.02	moderate	BC-CH 25	0.73 ± 0.03	moderate
BC-YG 6	0.33 ± 0.01	weak	BC-CH 26	0.86 ± 0.04	moderate
BC-CT 7	1.37 ± 0.12	strong	BC-CH 27	1.10 ± 0.03	strong
BC-CT 8	0.45 ± 0.02	weak	BC-CH 28	0.88 ± 0.01	moderate
BC-CT 9	0.44 ± 0.03	moderate	BC-CH 29	1.21 ± 0.02	strong
BC-CT 10	0.8 ± 0.02	moderate	BC-CH 30	1.01 ± 0.03	moderate
BC-CT 11	0.70 ± 0.02	moderate	BC-CH 31	1.10 ± 0.09	strong
BC-PW 12	0.54 ± 0.01	moderate	BC-RW 32	0.71 ± 0.02	moderate
BC-PW13	0.65 ± 0.03	moderate	BC-RW 33	0.71 ± 0.01	moderate
BC-RI 14	0.44 ± 0.04	weak	BC-RW34	1.21 ± 0.02	strong
BC-RI 15	0.66 ± 0.01	moderate	BC-RW35	1.85 ± 0.01	strong
BC-RI 16	0.30 ± 0.01	weak	BC-RW 36	1.43 ± 0.03	strong
BC-RI 17	0.98 ± 0.00	moderate	BC-SL 37	0.61 ± 0.03	moderate
BC-RI 18	1.13 ± 0.09	strong	BC-SL 38	1.23 ± 0.04	strong
BC-TF 19	1.64 ± 0.02	strong	BC-SL 39	1.01 ± 0.02	strong
BC-RI 20	0.74 ± 0.02	moderate	BC-BD 40	0.60 ± 0.01	moderate

SD, standard deviation. Strong: 14 (35%), moderate: 19 (47.5%), weak: 7 (18%).

## Data Availability

The data presented in this study are available upon request.

## Supplementary Materials

The following supporting information can be downloaded at: https://www.mdpi.com/article/10.3390/antibiotics13080774/s1, Table S1: oligonucleotide primers for detection of specific antimicrobial resistance genes; Table S2: growth of *B. cereus s. s.* isolates at 7 °C/7 days; Table S3: MLST profiles of *B. cereus s. s.* isolates; Table S4: oligonucleotide primer sequences, target genes, and cycling conditions used in this study; Table S5: The antimicrobial disc concentrations and interpretative standard zone diameters used for the disk diffusion test of *B. cereus s. s.;* Figure S1: Distribution of enterotoxin and cereulide genes in *B. cereus s. s.* isolates. References [73,93,94,103,104,105,106,107,108,109,110,111,112,113] are cited in the supplementary materials.
