# Supplementary material for: Genetic Characterization, Antibiotic Resistance, and Virulence Genes Profiling of Bacillus cereus Strains from Various Foods in Japan
Source: Antibiotics (Basel). 2024 Aug 16;13(8):774. doi: 10.3390/antibiotics13080774 (PMC11351997; doi:10.3390/antibiotics13080774)
Supplement: Supplementary file 1 [file antibiotics-13-00774-s001.zip › antibiotics-3139888-supplementary.pdf]

Table S1: Oligonucleotide primers for detection of specific antimicrobial resistance genes

Gene	Primer sequence (5′-3′)	Product size (bp)	Annealing temperat-ure (°C)	References
tetA	F: GGCGGTCTTCTTCATCATGC<br>R: CGGCAGGCAGAGCAAGTAGA	502	58	[73]
erm	F: TCTAAAAAGCATGTAAAAGAA<br>R: TTCGATAGTTTATTAATATTAGT	645	52	[112]
*van*A	F: ATTGCTATTCAGCTGTACTC<br>R: GGCTCGAGTTCCTGATGAAT	559	57	[113]
*van*B	F: AACGGCGTATGGAAGCTATG<br>R: CCATCATATTGTCCTGCTGC	467	57	[113]
*van*R	F: ATTAAGAAGGGCAGCAACGA<br>R: TTTGTAGCCAATGCCTTTCA	310	55	This study
*van*S	F: AGGCAGAACAAGCAAAAAGC<br>R: GGAAAAAGGGACGACAATCA	327	55	This study
*van*Y	F: GATGGGTATAGACCGCCAGA<br>R: TCGTTTCCCCAAAAACTGTC	337	55	This study
*van*W	F: TTGCTGGGGGCATATTACTT<br>R: ATTCACCGTTTTCAGCTTGC	345	55	This study
*van*H	F: CTACAGGGGTTTGGAGGTCA<br>R: CTACAGGGGTTTGGAGGTCA	370	55	This study

Table S2: Growth of *B. cereus s. s.* isolates at 7 °C/ 7 days:

Strain code	OD values (mean ± SD)				Strain code	OD values (mean ± SD)			
	Day 0	Day 3	Day 5	Day 7		Day 0	Day 3	Day 5	Day 7
BC-UH 1	0.004 ± 0.002	0.159 ± 0.050	0.290 ± 0.038	0.545 ± 0.108	BC-TF 21	0.005 ± 0.001	0.165 ± 0.047	0.239 ± 0.066	0.367 ± 0.086
BC-UH 2	0.003 ± 0.001	0.162 ± 0.006	0.344 ± 0.068	0.655 ± 0.110	BC-TF 3	0.006 ± 0.002	0.130 ± 0.018	0.671 ± 0.440	0.253 ± 0.070
BC-PM 3	0.004 ± 0.002	0.227 ± 0.038	0.397 ± 0.133	0.871 ± 0.046	BC-CH 23	0.007 ± 0.002	0.160 ± 0.004	0.224 ± 0.069	0.265 ± 0.131
BC-PM 4	0.006 ± 0.002	0.174 ± 0.009	0.329 ± 0.055	0.838 ± 0.077	BC-CH 24	0.006 ± 0.001	0.171 ± 0.041	0.165 ± 0.083	0.355 ± 0.128
BC- Yg5	0.006 ± 0.001	0.274 ± 0.048	0.305 ± 0.052	0.782 ± 0.127	BC-CH 25	0.006 ± 0.002	0.059 ± 0.042	0.206 ± 0.042	0.389 ± 0.075
BC- YG 6	0.006 ± 0.001	0.163 ± 0.033	0.166 ± 0.058	0.311 ± 0.090	BC-CH 26	0.006 ± 0.001	0.157 ± 0.058	0.271 ± 0.028	0.314 ± 0.025
BC-CT 7	0.007 ± 0.002	0.220 ± 0.044	0.352 ± 0.051	0.537 ± 0.072	BC-CH 27	0.007 ± 0.001	0.301 ± 0.013	0.497 ± 0.030	0.852 ± 0.073
BC-CT 8	0.004 ± 0.002	0.138 ± 0.010	0.271 ± 0.045	0.557 ± 0.112	BC- CH 28	0.005 ± 0.001	0.116 ± 0.024	0.261 ± 0.059	0.416 ± 0.086
BC-CT 9	0.004 ± 0.001	0.103 ± 0.006	0.233 ± 0.058	0.530 ± 0.118	BC-CH 29	0.007 ± 0.002	0.145 ± 0.009	0.22 ± 0.031	0.360 ± 0.054
BC-CT 10	0.006 ± 0.003	0.131 ± 0.040	0.332 ± 0.051	0.742 ± 0.057	BC-CH 30	0.005 ± 0.001	0.075 ± 0.005	0.275 ± 0.042	0.439 ± 0.074
BC-CT 11	0.006 ± 0.001	0.095 ± 0.022	0.172 ± 0.034	0.211 ± 0.038	BC-CH 31	0.004 ± 0.001	0.291 ± 0.021	0.807 ± 0.034	0.902 ± 0.054
BC-PW 12	0.006 ± 0.001	0.175 ± 0.013	0.259 ± 0.105	0.234 ± 0.056	BC- RW 32	0.006 ± 0.002	0.251 ± 0.045	0.589 ± 0.041	0.907 ± 0.007
BC-PW13	0.008 ± 0.000	0.156 ± 0.034	0.188 ± 0.029	0.443 ± 0.062	BC-RW 33	0.007 ± 0.001	0.088 ± 0.011	0.255 ± 0.066	0.651 ± 0.078
BC-RI 14	0.006 ± 0.001	0.104 ± 0.026	0.354 ± 0.010	0.723 ± 0.042	BC-RW34	0.005 ± 0.001	0.051 ± 0.014	0.373 ± 0.096	0.907 ± 0.039
BC-RI 15	0.007 ± 0.002	0.216 ± 0.028	0.205 ± 0.014	0.651 ± 0.051	BC-RW35	0.004 ± 0.001	0.275 ± 0.357	0.271 ± 0.123	0.707 ± 0.023
BC-RI 16	0.008 ± 0.001	0.233 ± 0.036	0.264 ± 0.066	0.488 ± 0.050	BC-RW 36	0.007 ± 0.002	0.150 ± 0.016	0.244 ± 0.086	0.421 ± 0.169
BC-RI 17	0.006 ± 0.001	0.215 ± 0.015	0.294 ± 0.044	0.816 ± 0.099	BC-SL 37	0.007 ± 0.001	0.136 ± 0.045	0.321 ± 0.017	0.758 ± 0.091
BC-RI 18	0.004 ± 0	0.123 ± 0.043	0.331 ± 0.085	0.649 ± 0.062	BC-SL 38	0.006 ± 0.001	0.167 ± 0.013	0.250 ± 0.048	0.925 ± 0.058
BC-TF 19	0.004 ± 0.001	0.077 ± 0.024	0.194 ± 0.009	0.448 ± 0.115	BC-SL 39	0.004 ± 0	0.22 ± 0.044	0.466 ± 0.069	0.587 ± 0.093
BC-RI 20	0.006 ± 0.002	0.167 ± 0.057	0.255 ± 0.075	0.368 ± 0.081	BC-BD 40	0.003 ± 0.001	0.092 ± 0.012	0.201 ± 0.011	0.256 ± 0.059

Table S3: MLST profiles of *B. cereus s. s.* isolates

Starin code	Source	MLST								
		glp	*gmk*	*ilv*	*pta*	*pur*	*pyc*	*tpi*	ST	ID in database
BC-PM 4	Pasturized milk	13	9	14	11	11	12	10	2860*	5398
BC-CT 8	Cheese	13	9	14	11	9	251	7	2867*	5397
BC-PW 12	Pasturized milk	3	2	14	5	16	3	4	2887*	5399
BC- CH 25	Chicken meat	13	8	9	11	4	12	7	2888*	5395
BC-CH 28	Chicken meat	13	8	326	11	11	12	7	2889*	5396
BC-RW 32	Raw milk	14	8	13	19	8	17	17	2891*	5400
BC-RW 35	Raw milk	22	8	49	220	4	3	4	2896*	5401
BC-CT 9	Cheese	14	17	13	19	2	17	7	2910*	5403
BC-CH 23	Chicken meat	47	8	43	11	387	36	7	2917*	5404
BC-UH 1	UHT milk	117	9	14	11	2	3	7	2918*	5391
BC-RI 20	Rice	14	9	9	260	2	17	7	2919*	5405
BC-RI 15	Rice	37	9	14	44	9	53	**344**	2920*	5406
BC-RI 16	Rice	3	2	14	17	36	3	7	2928*	5407
BC-RI 17	Rice	38	9	14	220	192	38	4	2929*	5408
BC-RI 18	Rice	117	9	9	260	2	27	7	2931*	5409
BC-CH 29	Chicken meat	11	9	14	12	206	14	7	2932*	5410
BC-TF 21	Tofu	3	9	326	11	58	3	7	2933*	5411
BC-CH 24	Chicken meat	37	9	256	4	2	6	136	2934*	5412
BC-RI 14	Rice	37	8	49	44	**387**	36	**344**	2899*	5402
BC-PM 3	Pasturized milk	50	9	14	12	19	3	7	2337	5417
BC-Yg 5	Yoghurt	14	8	13	19	2	17	7	1207	5418
BC-SL 38	Farm soil	14	8	13	11	2	17	17	1274	5419
BC-CT 7	Cheese	11	9	14	29	232	27	7	1243	5420
BC-CT10	Cheese	14	8	13	5	2	17	7	2528	5421
BC-PW 13	Pasturized milk	198	9	18	220	192	38	7	1182	5422
BC-CH 26	Chicken meat	14	8	13	11	2	17	17	1274	5423
BC-SL39	Farm soil	13	8	8	11	11	12	10	999	5424
BC-SL37	Farm soil	65	1	293	1	1	53	19	2261	5425
BC-BD40	Cow bedding	65	1	293	1	1	53	42	1887	5426
BC-TF3	Tofu	3	9	9	186	1	37	**345**	2937*	5429
BC-RW34	Raw milk	3	9	9	11	230	17	7	2936*	5430
BC20 *B. cereus*	Food manufacturing facility	3	2	31	5	16	3	4	26	5414
B. cereus K-F	Food manufacturing facility	3	2	14	5	16	3	4	2887*	5415
B.cereus M No.5	Dairy product	22	9	18	220	20	19	7	1063	5416
BC22 *B. cereus*	Food manufacturing facility	3	9	14	5	230	3	4	2938*	5427
B.cereus M No.4	Pudding	22	9	14	5	16	3	4	2941*	5428

Novel STs found in this study were marked with an asterisk (*).
Novel Alleles found in this study were marked in Bold.

Table S4: Oligonucleotide primer sequences, target genes, and cycling conditions used in this study:

The Target Gene	Primer	Primer sequence (5′-3′)	Product size (bp)	Annealing temperature (°C)	Reference
gyrB	BC1<br>BC2r	ATTGGTGACACCGATCAAACA<br>TCATACGTATGGATGTTATTC	365	56	[93]
gyrB	BT1<br>BT2	ATCGGTGATACAGATAAGACT<br>CCTTCATACGTATGAATATTATTT	368	56	[93]
cry	K3<br>K5	GCTGACACGAAGGATATAGCCAC<br>AGGACCAGGATTTACAGGAGG	1,600-<br>1,700	52	[103]
hblA	HblA1<br>HblA2	GCTAATGTAGTTTCACCTGTAGCAAC<br>AATCATGCCACTGCGTGGACATATAA	874	58	[104]
hblC	HBLC-N<br>HBLC-C	AATAGGTACAGATGGAACAGG<br>GGCTTTCATCAGGTCATACTC	400	62	[105]
hblD	HBLD-N<br>HBLD-C	AATCAAGAGCTGTCACGAAT<br>CACCAATTGACCATGCTAAT	439	54	[106]
nheA	nheA-F<br>nheA-R	TACGCTAAGGAGGGGCA<br>GTTTTTATTGCTTCATCGGCT	500	55	[107]
nheB	NBF<br>NBR	TTTAGTAGTGGATCTGTACGC<br>TTAATGTTCGTTAATCCTGC	743	54	[108]
nheC	NCF<br>NCR	TGGATTCCAAGATGTAACG<br>ATTACGACTTCTGCTTGTGC	683	54	[109]
bceT	ETF<br>ETR	TTACATTACCAGGACGTGCTT<br>TGTTGTGATTGTAATTTCAGG	428	58	[111]
entFM	ENT-A<br>ENT-B	ATGAAAAAAGTAATTTGCAGG<br>TTAGTATGCTTTTGTGTAACC	1,269	52	[110]
cytK	F2<br>R7	AACAGATATCGGTCAAAATGC<br>CGTGCATCTGTTTCATGAGG	623	55	[111]
ces	CesF1<br>CesR2	GGTGACACATTATCATATAAGGTG<br>GTAAGCGAACCTGTCTGTAACAACA	1,271	52	[94]
cesA	Ces-AF<br>Ces-AR	AAGATTGTCGCATATGTTGTG<br>TTATTAAGAGGCAATGCCTTC	188	55	This study
PCR Conditions		step1:95°C, 2min.<br>step 2: 94°C, 60 s.<br>step 3: annealing temperature (above), 60-90 s.<br>step 4: 72 °C, 60 s.<br>step (2- 4), 30 cycles.<br>step 5: 72 °C, 7 min.			

Table S5: The antimicrobial disk concentrations and interpretative standard zone diameters used for the disk diffusion test of *B. cereus s. s.*

Antibiotics	Disc Concentration	Zone diameter (mm)		
		Resistant (≤)	Intermediate	Susceptible(≥)
Ampicillin (ABP)	10 μg	28	-	29
Amoxicillin-Clavulanic acid (ACV)	20μg/10 μg	19	-	20
Cefepime(CFP)	30 μg	14	15-22	23
Cefoxitin (CFX)	30 μg	19	20-24	25
Oxacillin(MPI)	10 μg	10	11-12	13
Penicillin (PC)	10 units	28	-	29
Gentamicin (GM)	10 μg	12	13-14	15
Kanamycin (KM)	30mg	13	14-17	18
Erythromycin (EM)	15mg	13	14-22	23
Vancomycin (VCM)	30mg	13	14	15
Tetracycline (TC)	30mg	14	15-18	19
Rifampicin (RA)	5mg	16	17-19	20
Trimethoprim-Sulfamethoxazole (ST)	1.25μg-23.75μg	10	11-15	16

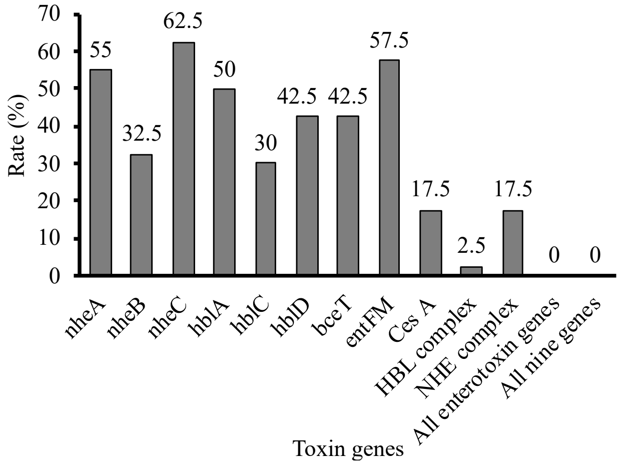

Figure S1 Distribution of enterotoxin and cereulide genes in *B. cereus s. s.* isolates
